# Effectiveness of Mobile Health Augmented Cardiac Rehabilitation on Clinical Outcomes among Post-Acute Coronary Syndrome Patients: A Randomized Controlled Trial

**DOI:** 10.12669/pjms.42.6.11481

**Published:** 2026-06

**Authors:** Aliya Hisam, Zia Ul Haq, Patrick Doherty, Jill Pell, Sohail Aziz

**Affiliations:** 1 Aliya Hisam, MBBS, MPH, CHPE, FCPS, PhD, Associate Professor, Department of Community Medicine, Army Medical College, National University of Medical Sciences, Rawalpindi, Pakistan; 2 Zia Ul Haq, MBBS, MPH, PhD, Professor, Vice Chancellor, Khyber Medical University, Peshawar, Pakistan; 3 Patrick Doherty, PhD, Professor, Department of Health Sciences, University of York, United Kingdom; 4 Prof Jill Pell, MBChB, MD, FFPH, Professor, Institutes of Health & Wellbeing, University of Glasgow, United Kingdom; 5 Prof Sohail, MBBS, FCPS, MRCP, MPhil, FSCAI, FRCP, Professor, Fauji Foundation Hospital, Rawalpindi, Pakistan

**Keywords:** Cardiac rehabilitation, Rehabilitation, Myocardial infarction, Heart attack, Acute coronary syndrome, Clinical outcomes, Blood pressure, Weight, Body Mass Index

## Abstract

**Objective:**

To determine the effectiveness of mobile health augmented cardiac rehabilitation on clinical factors among post-acute coronary syndrome (post-ACS) patients.

**Method:**

A randomized controlled trial was conducted at Armed Forces Institute of Cardiology, Rawalpindi. This study was conducted on inpatients post-ACS patients. Patients were randomly allocated into the intervention group (counselling, short text messages, and standard post-ACS care) or the control group (standard post-ACS care). Clinical factors assessed were blood pressure, body mass index, the number of days of hospitalization, no of readmissions, and major adverse clinical events (MACE) within 180 days post-ACS. Data were collected thrice for six months follow-up periods (baseline, 12 and 24 weeks).

**Result:**

The mean days of hospitalization were reduced significantly of the intervention group compared to the control group (02.55, 95%CI: 02.13, 02.98 versus 04.07, 95%CI: 02.96, 05.17, p-value=0.033) at 12 weeks follow up. Also, at 24 weeks follow up, the intervention group reduced the number of readmission days compared to the control group (02.62, 95% CI: 02.00, 03.24, versus 03.71, 95%CI: 02.68, 04.74, p-value=0.041). Twenty-nine (43.94%) patients of the control group and 18 (24.32%) of the intervention group developed MACE at 12 weeks follow up (p-value: 0.016). While at 24 weeks follow up, 8 (16.33%) of the control group and 7 (9.86%) of the intervention group suffered MACE (p-value: 0.196).

**Conclusion:**

The MCard positively impacts the clinical outcomes. It has significantly decreased the likelihood of readmission and decreased the number of days of hospitalization among the MCard group compared to the usual care group. This trial has found an overall lower incidence of MACE (composite of readmission, myocardial re-infarction, heart failure and death) among the MCard group than usual care.

***Registration:*** Australian New Zealand Clinical Trial Registry (ANZCTR) (ACTRN12619001731189).^16^

## INTRODUCTION

Acute coronary syndrome (ACS) has three traditional forms that are non-ST-elevation myocardial infarction (NSTEMI), ST-elevation myocardial infarction (STEMI), and unstable angina.^1^ Patient with ACS has a higher risk of recurrence clinical problems in the long run as re-hospitalizations after ACS are more common in patients with chronic diseases as diabetes, hypertension. When the number of associated comorbidities in ACS grows, so does the Risk of morbidities and mortalities.^2^ To avoid readmission and recurring ACS, guidelines suggest offering an evidence-based post-discharge plan that includes cardiac rehabilitation (CR), treatment, patient/caregiver education, and ongoing follow-up.^3^

Management of modifiable risk factors as blood pressure (BP), body mass index (BMI) and low-density lipoprotein revealed that one year after an ACS, the 10-year Risk of repeated cardiovascular events decreases to almost 20 per cent. Considerable delays in care-seeking are common in patients with ACS, which counteracted by one of the components of CR in which patients are counselled for regular follow-up visits to hospitals.^4^-^6^

The CR program is beneficial in improving patient outcomes and is still underutilized by post-ACS patients. To address this low utilization, mHealth provides an alternative method that aims to improve CR program uptake and complete utilization. Arthur et al. conducted a study in which he investigated home-based CR to Hospital-based CR and found that the attendance of HB CR was more than double compared to a hospital-based program over 24 weeks.^7^ Disturbed physiological parameters such as high BMI, increased BP, impaired lipid levels are widespread among ACS patients and is linked to an increased risk of death and cardiovascular events.^8^

There is also evidence of BMI correlation with the increased incidence of MACE, which is associated independently. A study has shown that women are more likely to have MACE than men.^9^ MACE is decreased in patients with heart failure who had percutaneous intervention after acute myocardial infarction when they received early CR.^10^ CR involving nutrition counselling effectively reduces BMI and BP.^11^ A trial showed that CR improved left atrial and ventricular function with a consequent reduction in BP, suggesting secondary prevention measures have been very effective in reducing MACE and improving physiological parameters.^12^

Acceptability is a difficult concept to define. The complexity of the idea is shown by the wide range of definitions found in the medical literature. The words “treatment acceptability” and “social acceptability” are specific examples of definitions. These phrases suggest that acceptability might be examined from an individual standpoint, but it can also reflect a more broadly shared assessment of an intervention’s character.^13^ Mobile Health (MHealth) systems are being used for patients with ACS by offering a home-based educational and self-management intervention.

These mHealth applications provide user-friendly resources for monitoring physiological and wellbeing parameters such as BP, weight and assisting patients in self-managing their condition.^14^ A systematic review and meta-analysis to look at the Impact of mobile-based health interventions on lowering cardio-metabolic Risk by encouraging physical activity and safe lifestyle choices revealed significant mean changes for BMI, waist circumference and BP.^15^

## METHODOLOGY

It was a superiority, parallel-group, two-arm, single-centre, randomized controlled study that spanned from 2019 to 2021. The Armed Forces Institute of Cardiology in Rawalpindi, Pakistan, was the site of the trial. Informed consent was obtained from patients with ACS who arrived at the emergency room and were admitted to their wards.

The trial is registered in the Australian New Zealand Clinical Trial Registry (ANZCTR) (ACTRN12619001731189)^16^ and complies with the CONSORT declaration (2010). The protocol has been published.^17^ One hundred and eighty post-ACS patients were divided into two groups at random (1:1) for this trial: the MCard intervention group and the control group. In addition to routine post-ACS care, the intervention group received the MCard intervention, a medically supervised CR program. Individualized psychotherapy was part of the MCard’s initial phase while the patient was in the hospital.

Daily mobile texting with standardized messages promoting adopting healthier lifestyles was part of the second phase. The only post-ACS care given to the control group was routine. Each participant received a self-monitoring device (a digital blood pressure monitor, a weight machine, and a pedometer) along with a pamphlet to record their results. Blood pressure, body mass index, the number of hospital days, the frequency of readmissions, and major adverse clinical events (MACE) within 180 days after ACS were among the clinical characteristics evaluated. The following behavioural characteristics were evaluated: medication compliance, smoking, salt intake, blood pressure, weight self-monitoring, physical activity (IPAQ tool), and healthy eating (Healthy Eating Assessment tool). A Likert scale was also used to gauge acceptability.

STATA 14 was used to enter and analyse the data. Chi-square tests were used to compare the two groups after categorical data were shown as frequencies and percentages. Where appropriate, independent sample t-tests were employed for comparisons, and the mean with 95% CI was displayed for continuous data. other statistical test was applied where required are mentioned under foot notes. A p-value of less than 0.05 was considered significant.

## RESULTS

The body mass index among the control and intervention group at baseline, 12 and 24 weeks has not been affected by the intervention significantly. At baseline, the BMI was 24.20 (23.23, 25.16) among control versus 25.34 (24.40, 26.28) in intervention group (p=0.092). At 12 weeks and 24 weeks, the mean BMI was different among control and intervention group (24.98, 95% CI: 23.88,26.09 versus 25.82, 95% CI:24.78, 26.86, p=0.092) (25.12, 95% CI: 24.01, 26.24 versus 26.10, 95% CI: 25.11, 27.09, p=0.196) respectively but this difference was not significant.

### Blood Pressure:

At 12 weeks as the systolic blood pressure among control was 123.96 (95%CI: 120.40, 127.51) and in intervention was 122.32 (95%CI: 119.19, 125.45) (p-value=0.495) and the diastolic blood pressure in control was 80.40 (95%CI:77.98, 82.81) and intervention was 78.66 (95%CI: 76.55, 80.76) (p-value=0.284). At 24 weeks follow up, the control group systolic blood pressure was 126.53 (95%CI:123.47, 129.59) and among intervention group it was 123.19 (95%CI: 120.29, 126.08) (p-value=0.123) and the diastolic blood pressure in control was 82.82 (95% CI: 95%CI 80.75, 84.88) and among intervention was 79.81 (95% CI: 77.89, 81.74) (p-value=0.039). Overall, there was a significant difference only in the diastolic blood pressure levels between the two study groups at both periods.

### Days of Hospitalization:

The mean days of hospitalization of the control and intervention group were 04.07 (95%CI: 02.96, 05.17) and 02.55 (95%CI: 02.13, 02.98) respectively at 12 weeks follow up. While at 24 weeks follow up, the control and intervention group mean the number of hospitalization days was 03.71 (95%CI: 02.68, 04.74) and 02.62 (95%CI: 02.00, 03.24), respectively. The number of days of hospitalization among the two groups at 12 and 24 weeks was significantly different (p-value=0.033, 0.041), respectively, showing some evidence of the effectiveness of MCard intervention on the number of days of hospitalization (Table-I).

**Table-I:** Anthropometric and physiological outcomes among post-ACS participants at baseline and follow-ups.

Variable	Baseline		12 weeks		24 weeks	
	Mean (95% CI)	p-value	Mean (95% CI)	p-value	Mean (95% CI)	p-value
***Body mass index***						
Control	24.20 (23.23, 25.16)	0.092	24.98 (23.88,26.09)	0.281	25.12 (24.01, 26.24)	0.196
Intervention	25.34 (24.40, 26.28)		25.82 (24.78, 26.86)		26.10, (25.11, 27.09)	
***Systolic blood pressure***						
Control	128.46 (122.91, 134.01)	0.379	123.96 (120.40, 127.51)	0.495	126.53 (123.47,129.59)	0.123
Intervention	125.45 (121.51, 129.38)		122.32(119.19, 125.45)		123.19 (120.29, 126.08)	
***Diastolic blood pressure***						
Control	81.31 (77.52, 85.11)	0.065	80.40 (77.98, 82.81)	0.284	82.82 (80.75, 84.88)	0.039
Intervention	77.25 (75.11, 79.39)		78.66 (76.55, 80.76)		79.81 (77.89, 81.74)	
***Days of hospitalization***						
Control	2.88 (02.57, 03.19)	0.884	04.07 (02.96, 05.17)	0.033	03.71 (02.68, 04.74)	0.041
Intervention	2.85 (02.44, 03.25)		02.55 (02.13, 02.98)		02.62 (02.00, 03.24)	

MACE=12 weeks follow-up: Control n=49, Intervention n=70 24 weeks follow-up: Control n=50, Intervention n=7.

### MACE:

**Fig.1:**
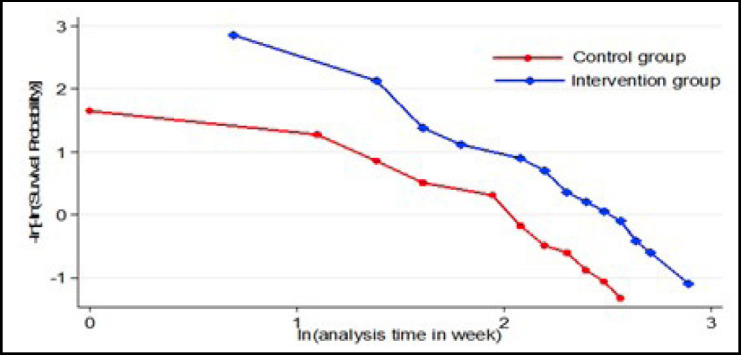
Proportional Hazard’s Assumption Testing by Log Survival Plot.

The incidence of MACE was significantly lower in the MCard group in comparison to the control group. The total MACE incidence during the whole study period was 61 (38.12%) among both groups. Twenty-nine (43.94%) patients of the control group and 18 (24.32%) of the intervention group developed MACE at 12 weeks follow up (p-value: 0.016). The normality for survival analysis, cox proportional was checked (Fig.1).

**Fig.2:**
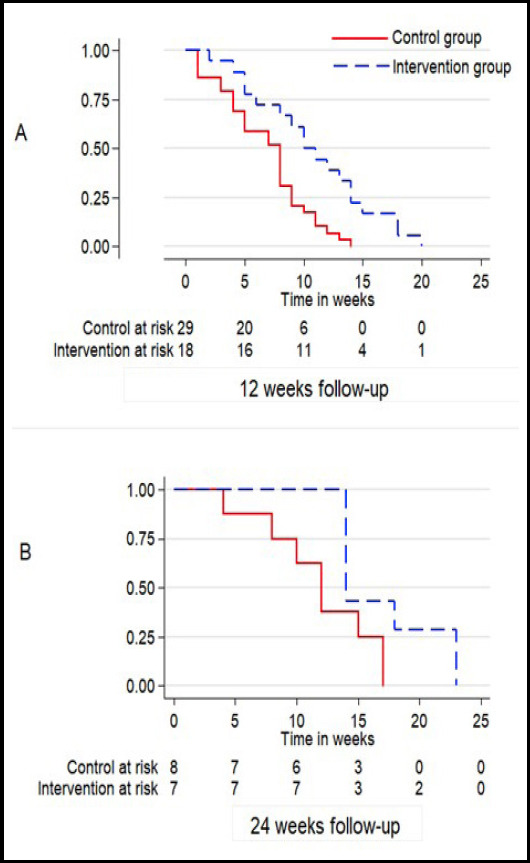
Plot Of Kaplan Meir Survival Estimate Of Mace Control (Red) And Mcard Group (Blue) (A) Mace Incidence At 12 Weeks (B) Mace Incidence At 24 Weeks.

While at 24 weeks follow up, 8 (16.33) of the control group and 6 (8.57%) of the intervention group suffered MACE (p-value: 0.196). Among the MACE variables at 12 weeks, deaths (control=17, 25.37%: intervention=4, 5.33%, p-value: 0.019) were significantly more in the control group than the. The MCard group has a much lower rate of readmissions, re-infarction, re-symptom, heart failure, and death when compared to the control group, but it was not significantly different. A total of 22 (17.7%) post-discharge deaths among the 160 participants occurred between baseline and 24 weeks follow up period. From baseline till 12 weeks’ period, there were 17 (25.37%) deaths among the control group while the intervention group had 5 (6.25%) deaths. Between 12 weeks till 24 weeks’ follow-up period, only 1 (1.43%) death occurred among the intervention group. (Table-II & Fig.2).

**Table-II:** Clinical Outcomes Of Mcard Participants At 12- And 24-Weeks Follow-Up.

	12 weeks follow-up (n=121)			24 weeks follow-up (n=119)		
	Yes	No		Yes	No	
	n (%)	n (%)	p-value	n%	n (%)	p-value
***Readmission***						
Control	18 (31.03)	40 (68.97)	0.170	8 (16.33)	41 (83.67)	0.196
Intervention	15 (20.55)	58 (79.45)		6 (8.57)	64 (90.14)	
***Re-infarction***						
Control	3 (5.36)	53 (94.64)	0.201	-	49 (100.00)	-
Intervention	1 (1.39)	71 (98.61)		-	70 (100.00)	
***Re-symptom***						
Control	12 (21.82)	43 (78.18)	0.893	9 (19.37)	40 (81.63)	0.113
Intervention	15 (20.83)	57 (79.17)		6 (8.57)	64 (91.43)	
***Heart Failure***						
Control	1 (1.79)	55 (98.21)	0.450	1 (2.04)	48 (97.96)	0.230
Intervention	3 (4.11)	70 (95.89)		-	70 (100.00)	
***Death***						
Control	17 (25.37)	50 (74.63)	0.001	-	49 (100.00)	0.401
Intervention	4 (5.33)	70 (94.59)		1 (1.41)	70 (98.59)	
***MACE***						
Control	29 (43.94)	37 (56.06)	0.016	8 (16.33)	41(83.67)	0.196
Intervention	18 (24.32)	55 (75.34)		7 (9.86)	64 (90.14)
***Lost to follow-up***						
Control	13 (16.25)	67 (83.75)	0.045	-	49 (100.00)	0.401
Intervention	5 (6.25)	75 (93.75)		1 (1.43)	69 (98.57)	

## DISCUSSION

CR services are multi-component, including initial evaluation, risk factor management, patient education, and exercise delivered through a specialist team. The primary purpose of CR is to bring lifestyle modifications and improve patient’s outcomes within a period.^18^ This randomized clinical trial was mobile health assisted CR, having 24 weeks’ follow-up and having three times data collection. From baseline to 12 weeks and then 24 weeks, there was an improvement in clinical parameters among the intervention group. MACE decreased incidence among the MCard group and decreased BP and days of hospitalization. Acceptability of the MCard measure was seen in the majority of the intervention group participants. There was a 10% lost to follow up.

This study showed a significant improvement in the blood pressure and MACE incidence among the intervention group compared to the control group at the end of the trial. While a similar study revealed, BMI did not affect survival or the risk of significant cardiovascular and bleeding complications in elderly patients with ACS.^19^ Another trial using mobile technology for CR found no significant difference in body weight and blood pressure taken as secondary outcomes and followed up to one year.^20^ Both of these studies contradict these study findings. There may be some methodological differences in the delivery of the mHealth intervention or CR components or some unexplored reasons, which need to be explored in future reviews.

In a study conducted by Mirman AM et al., an intensive CR program was compared with traditional CR program showing BMI, body fat percentage, BP, and cholesterol levels, all substantially improved in both types of CR programs, indicating that CR is effective in improving clinical parameters whether it is a traditional or an advanced CR program.^21^ Another research showed that BMI was correlated with the incidence of MACE negatively and independently.^22^ A 12-week Phase-CR program revealed a reduction in BMI, BP and LDL-C levels, especially obese individuals.^23^ It has also been shown that MACE risk is higher in individuals with versus without Heart Failure following PCI for ACS.^24^ Another research revealed post-ACS patients who did not complete a standard 12-week CR program had a higher long-term occurrence of adverse cardiac events than those who completed the program.^25^

Rosario MBD et al. conducted a pilot study in which patients were empowered with self-monitoring devices that are weight machines and BP apparatus, same as in this study, but gave a smartphone device to sixty-six patients.^26^ The change in the systolic BP among the control and intervention group from baseline to 24 weeks was 3.84 + 3.01 and -2.97 + 3.33, p=0.11, which is almost the same as in this study that is 1.93 + -0.56 and 2.26 + 5.16, p=0.001. The diastolic BP change was 1.88 + 1.76 and -0.64 + 1.52, p=0.38, while in this study it is -1.51 + 3.23 and -2.56 + 2.78 in control and intervention respectively. Mitchael et al. also reported the mean change in body weight in kg of control and intervention that is 0.56 + 0.26 versus -1.17 + 0.28.

In this study, BMI is reported instead of only weight. That is the mean change in BMI is -0.92 + -0.78 and -0.76 + -0.71, p=0.001. There was a significant change in BP and weight and BMI among both studies, indicating mHealth intervention incorporated in CR brings a significant change in physiological parameters. At the end of the trial, the completion rate of the intervention group participants at the end of the trial, that 24 weeks was 88%, compared to the 90% completion rate of this study. This confirms that utilization of CR programs that are mHealth augmented is significantly more than traditional hospital-based CR programs.

This MCard intervention has not supplied smartphone devices to the individuals as only text messages were communicated to the participants. However, in addition to the weight scale and BP apparatus, participants were given a pedometer to monitor and report the steps taken 24 hours on the brochure every week. As mentioned earlier in the behavioural outcomes, this had increased their physical activity level, contributing to the lower BMI in the results of this clinical outcome.

## CONCLUSION

The MCard positively impacts the clinical outcomes as it has decreased the BP and decreased the number of days of hospitalization among the MCard group compared to the control group. This trial has found an overall lower incidence of MACE (composite of readmission, myocardial reinfarction, heart failure and death) among the MCard group compared to the control group at the end of follow-ups. This suggests that the MCard intervention can alter the clinical spectrum of post-ACS patients cost-effectively and efficiently.

### Authors’ Contribution:

**AH and ZUH:** Perceived the objectives, literature review, design, and worked on methodology, and result writeup, data analysis, discussion and final drafting of manuscript.

**PD, JP & SA:** Performed the study, methodology approval, final result analysis and conducted a critical analysis for final approval and main scientific content.
